# The Response of Red Clover (*Trifolium pratense* L.) to Separate and Mixed Inoculations with *Rhizobium leguminosarum* and *Azospirillum brasilense* in Presence of Polycyclic Aromatic Hydrocarbons

**DOI:** 10.3390/ijerph17165751

**Published:** 2020-08-09

**Authors:** Karolina Furtak, Karolina Gawryjołek, Anna Gałązka, Jarosław Grządziel

**Affiliations:** Department of Agricultural Microbiology, Institute of Soil Science and Plant Cultivation State Research Institute, Czartoryskich 8, 24-100 Pulawy, Poland; kgaw@iung.pulawy.pl (K.G.); agalazka@iung.pulawy.pl (A.G.); jgrzadziel@iung.pulawy.pl (J.G.)

**Keywords:** *Azospirillum* sp., PAH, pollution, *Rhizobium* sp., symbiotic bacteria, *Trifolium pratense* L.

## Abstract

This study aimed to evaluate the impact of co-inoculation *Rhizobium* sp. and *Azospirillum* sp. on plant (*Trifolium pratense* L.) growth in the presence of polycyclic aromatic hydrocarbon (PAH) contamination (anthracene, phenanthrene, and pyrene). Eight strains from the genus *Rhizobium leguminosarum* bv. *trifolii* were selected for biotest analysis. Two methods of inoculation were used in the chamber experiment: (1) *R. leguminosarum* alone and (2) a combined inoculant (*R. leguminosarum* and *Azospirillum brasilense*). For comparison, non-contaminated controls were also used. The results demonstrated that co-inoculation of plants with *Rhizobium* and *Azospirillum* resulted in more root and shoot biomass than in plants inoculated with *R. leguminosarum* alone. The results indicated that application of a co-inoculation of bacteria from *Rhizobium* and *Azospirillum* species had a positive effect on clover nodulation and growth under the condition of PAH contamination.

## 1. Introduction

Polycyclic aromatic hydrocarbons (PAHs) are a group of over 200 compounds containing between two and several dozen aromatic rings per molecule. Their occurrence in the environment is mainly related to human activity; natural sources such as crude oil and volcanic eruptions also supply a small amount of them [1]. PAHs are accumulated in algae, invertebrates, and aquatic plants and are then transferred to higher trophic levels [2]. The accumulation of these contaminants in plant tissues poses a threat. As a result, PAHs are transmitted to herbivorous organisms, i.e., animals and humans [2]. The concentration and type of PAH in the soil can have an impact on plant biomass yield, plant growth, and biodegradation processes [3]. Some pollutants could also be genotoxic to plants [4]. Therefore, it is important to study the problem of PAH contamination in the context of its impact on plants and the effects of various groups of microorganisms on the growth rate and soil quality and plants in contaminated areas.

Plants play a very important role in the absorption and accumulation of organic pollutants [5]. The literature indicates that significant dissipation of PAH occurs in soils with grasses and legumes [6]. The plant species influences the mechanism of PAH dissipation and the level of absorption and adsorption of PAH from the environment. Depending on the species of plant, different species of microorganisms can also be found in the soil [7]. Root exudates from plants also influence the biodegradation process of PAHs [8]. It was found that the rate of degradation of pyrene and benzo[a]pyrene changed depending on the organic carbon content of clover root exudate [9]. It has been shown that endophytic bacteria can increase the resistance of plants to contamination and stimulate their protection mechanisms. Plant inoculation with endophytic bacteria that have the ability to use PAHs as a carbon source can promote bioremediation processes [10]. However, the presence of plants may also accelerate the biodegradation of PAHs via the stimulation of rhizosphere microorganisms by root exudates [11]. Phytoremediation supported by microorganisms can be the most effective way of biodegrading organic pollutants in soil. The research has demonstrated that the degradation of pyrene in soils planted with white clover (*Trifolium repens*) was significantly higher than in soils without plant. At the same time, clover did not grow less in the presence of pyrene compared to clover on uncontaminated soil [12].

Microorganisms play an important role in the degradation of PAHs in the environment and, due to their potential, they are the basis of bioremediation processes. Microbiological degradation of PAHs in soil depends on many factors. These include the bioavailability of compounds, the availability of nitrogen and phosphorus, aeration, and soil moisture [13]. Microorganisms decomposing PAHs belong to *Mycobacterium* sp., *Pseudomonas* sp., *Stenotrophomonas* sp., and *Burkholderia* sp. [6]. *Rhizobium* bacteria are also present in contaminated environments. Some species may use PAHs as an energy source. Furthermore, they have the potential to form symbiotic associations with leguminous plants [14]. *Rhizobium* possesses the biochemical and ecological capacity to degrade organic pollutants and can stimulate the other biodegrading bacteria in soil [15,16]. Johnson et al. [17] suggested that some strains of *R. leguminosarum* bv. *trifolii* can be relatively resistant to chryzene in soil. *R. leguminosarum* bv. *trifolii* can also establish symbiosis with *Trifolium* spp. plants and participate in the process of binding nitrogen [18].

Alpha-proteobacteria, including *Azospirillum* bacteria, participate in the early phases of PAH biodegradation [13]. Bacteria of the genus *Azospirillum* have the ability to nitrogen fix and are considered to be facultative endophytes. *Azospirillum* sp. can have a positive effect on the growth and survival of plants. Studies have shown that *A. brasilense* under spring wheat cultivation may cause a 10% increase in yield [19]. *A. brasilense* has also been successfully isolated from soil contaminated with coal tar and diesel [20]. The ability of *Azospirillum* sp. to degrade phenols and benzoates has also been demonstrated [21]. Research shows that *A. brasilense* is capable of hydrocarbon and PAH degradation [22,23]. Research carried out in the Department of Agricultural Microbiology IUNG-PIB has also shown that *Azospirillum* sp. strains can use energy from pyrene and anthracene decomposition in the process of nitrogen fixation [24,25]. 

Due to the usually poor nutrient availability in contaminated soils, much research has been conducted on the use of legumes, which fix nitrogen, and also nitrogen-fixing bacteria such as *Azospirillum* spp. *Azospirillum* spp. are nitrogen-fixing organisms (diazotrophs) that are capable of forming an associative relationship with the roots of several economically important cereals. They use PAHs as their only carbon and energy source, as well as produce biosurfactants [26]. *Rhizobium* spp. colonize the roots of legumes where they fix atmospheric nitrogen, some of which can be utilized for plant growth [18]. *Rhizobium* spp. are found in contaminated environments where various toxic chemicals are present. Rhizobia also have a positive effect on plants. They relieve the impact of nutrient deficiency in the soil on the plant and stimulate root growth and root exudation. The strain *R. leguminosarum* bv. *trifolii* KO17 was used to produce biofertilizer, which stimulated the germination and growth of legumes [27]. It was shown that *Rhizobium* had a positive effect on clover vigor and growth. The root density and the shoot weight were higher compared to clover that was not inoculated with *Rhizobium*.

Based on data obtained from the literature, we decided to check whether co-inoculation of red clover with a mixture of *Rhizobium leguminosarum* and *Azospirillum brasilense* would result in higher root nodulation and plant growth. The study aimed to evaluate the impact of co-inoculation with *Rhizobium* sp. and *Azospirillum* sp. on red clover (*Trifolium pretense* L.) growth in the presence of PAH contamination. The research hypothesis assumes that red clover inoculation with mixed inoculants of *Azospirillum* sp. and *Rhizobium* sp. may have a positive effect on plant nodulation and growth under the condition of PAH contamination. It was expected that the synergistic effect of both bacteria would reduce the toxic effects of PAH on the plant. These investigations are the first step in an analysis of the impact of inoculation with different cultures of microorganisms of plants growing in the presence of PAH contamination.

## 2. Materials and Methods 

### 2.1. Bacterial Strains and Media

The study used eight strains of *Rhizobium leguminosarum* bv. *trifolii* and three strains of *Azospirillum brasilense* bacteria from the collection of the Department of Agricultural Microbiology, Institute of Soil Science and Plant Cultivation in Puławy, Poland. Bacteria of the *Rhizobium* genus were isolated from root nodules derived from plants of the genus *Trifolium* and stored at 4 °C on Thornton’s medium [28]. Bacteria of the *Azospirillum* genus were isolated from the rhizosphere of spring barley and stored at 4 °C on PDA (potato dextrose agar) medium with the addition of malic acid [29]. Based on the sequencing of PCR products, we confirmed that bacteria strains belonged to *Rhizobium leguminosarum* and *Azospirillum brasilense* species. 

For *Rhizobium* bacteria, Thornton’s liquid medium was prepared, into which bacteria were transferred from the solid medium. Flasks were incubated at 28 °C in an orbital shaking incubator. After 5 d of incubation, liquid culture of *Rhizobium* bacteria containing 10^10^ bacteria per 1 mL was prepared for the experiment. The number of microorganisms was determined by the plate dilution method. 

Bacteria of the *Azospirillum* genus were transferred from solid PDA medium to liquid Nfb (nitrogen-free semisolid) medium [30] and cultured for 3 d at 28 °C in an orbital shaking incubator. *Azospirillum* culture containing 10^6^ bacteria per 1 mL of culture was used in the experiment.

### 2.2. PCR and Sequencing

For PCR, a small amount of material was taken from a single bacterial colony and transferred to a sterile eppendorf with 20 µl of MiliQ water. The samples were thoroughly mixed and 1 µl was taken from the mixture for the PCR reaction. The PCR conditions were as follows: initial denaturation (98 °C, 2 min), 35 cycles of denaturation (98 °C, 30 s), annealing (60 °C, 20 s), extension (72 °C, 90 s), and final extension (72 °C, 7 min) using CloneID (Lucigen, USA) polymerase ready-to-use mixture. The 16S rDNA region was amplified using primers: 27F (AGAGTTTGATCCTGGCTCAG) and 1492R (GGTTACCTTGTTACGACTT) [31]. The PCR products were sequenced in Genomed S.A (Warsaw, Poland) using the same primers as at the PCR step. The sequences from both primers were assembled in Unipro UGENE 1.25 software [32] and the BLAST search has been taken [33].

### 2.3. Metabolic Potential Assay 

The metabolic characteristics of *Rhizobium* sp. strains were determined using the GEN III™ Biolog method (Biolog Inc., Hayward, CA, USA). The GEN III™ microplate contains 94 phenotypic tests: 71 carbon source utilization assays and 23 chemical sensitivity assays. It provides a profile with a broad range of Gram-positive and Gram-negative bacteria. Tetrazolium dyes from the wells of the microplate are used to indicate the use of carbon sources or resistance to inhibitory chemicals by microorganisms. Among the compounds present on the GEN III™ plate, three groups of carbon substrates can be distinguished: carbohydrates (CH), amino acids (AA), and fatty acids (FA). Isolates of eight strains of *Rhizobium* sp. and three strains of *Azospirillum* sp. were prepared on agar medium and then suspended in an inoculating fluid (IF, Biolog Inc.). The cell suspensions were inoculated into the GEN III™ (100 μl per well) and incubated at 25 °C for 7 d. The intensity of color development was recorded at λ = 590 nm at 24 h intervals for a period of 168 h. The results obtained at 168 h are presented because the most intensive substrate decomposition was observed after this incubation time.

### 2.4. Biotest with Red Clover

**Figure 1:**
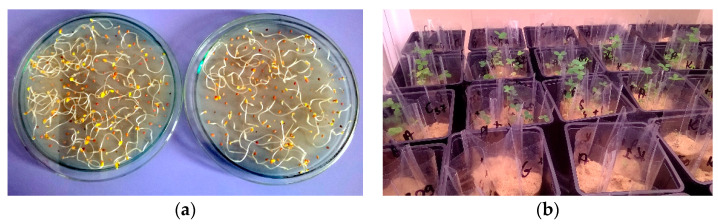
Biotest with clover: (**a**) growing clover sprouts on Jensen’s medium (1942) [44] before planting in sand; (**b**) clover growth on sand contaminated with PAH and inoculated with bacteria.

The experiment was conducted in controlled conditions in a chamber during the four weeks of plant growth. For the biotest, red clover (*Trifolium pratense* L.) was used. It is a herbaceous species of flowering plant in the bean family Fabaceae. The clover was selected because it grows relatively fast, has root nodules, and is a plant which is commonly found in Poland. The clover seed was sterilised in 3% perhydrol and washed five times with sterile water. The sprouts were cultured under sterile conditions on Jensen’s agar medium (Figure 1a) [34]. Sterile sand was placed in sterile plastic cells (100 g of sand each) and three cells were placed in one pot. Sterile sand was used to eliminate the influence of soil factors (microbiome, organic matter content, etc.) on interactions between the clover and the dosed bacteria. Moreover, the sand is poor in substances influencing the growth of the plants, so it was possible to control the number of nutrients applied with the Jensen medium and the contamination with hydrocarbons as the only components absorbed by the clover from the ground. Sand has successfully been used in experiments analysing PAH contamination [35,36,37]. 

Sand in cells in one pot was contaminated with three doses of a single PAH. The experiment used phenanthrene, anthracene, and pyrene (Table 1) in doses of 100 mg kg^−1^–0.01%, 500 mg kg^−1^–0.05% and 1000 mg kg^−1^–0.1%. The dose of 100 mg kg^−1^ is the limit for PAH in soils in industrial areas (according to German recommendations) [38]. The value of 500 mg kg^−1^ corresponds to the limit of PAH for soils used for agricultural purposes, above which there is a need for reclamation, while 1000 mg kg^−1^ is the limit for recreational areas (according to UK recommendations) [39]. PAHs were purchased from Sigma-Aldrich (Saint Louis, Missouri, USA). PAHs were added to the sand in liquid form (dissolved in dichloromethane; CH_2_Cl_2_) [40]. Next, the sand was stored for 24 h to allow for solvent evaporation.

The sand moisture content was maintained at a level of 60% field capacity (FC) [41] with Jensen’s liquid medium. Next, clover sprouts were planted on the prepared sand, with three replicates in each cell (Figure 1b). 

**Figure 2:**
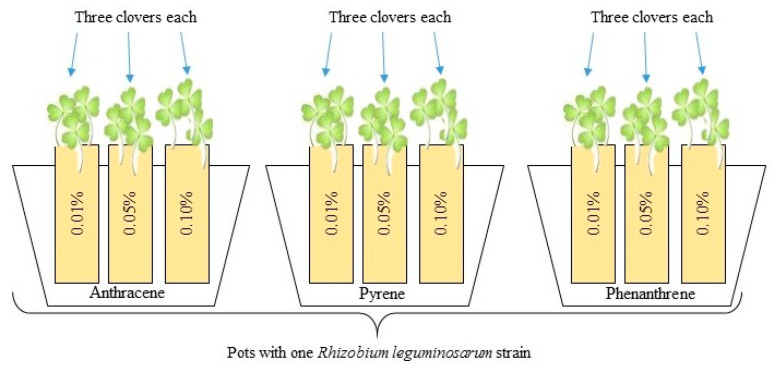
Scheme of biotest: each strain of *Rhizobium leguminosarum* was prepared with three pots, each containing sand contaminated with PAHs in three doses. Such pots were prepared in two repetitions—the second one was additionally co-inoculated with *Azospirillum brasilense*; three clovers were growing in each pot.

All plants were inoculated with the liquid culture of *Rhizobium leguminosarum* bacteria (Figure 2) in a volume of 1 mL per cell using a sterile pipette. In two pots with clover for all PAH doses, one strain of *Rhizobium* sp. was added. After three days, half of the prepared plants with different *Rhizobium* sp. strains were additionally inoculated with three mixed strains of *Azospirillum brasilense* (1 mL per cell). *Rhizobium*-inoculated clover was the control for plants inoculated with the mixed inoculants of *Azospirillum* sp. and *Rhizobium* sp. Additionally, 13 pots with clover growing without PAH contamination but inoculated with each *R. leguminosarum* strain were prepared.

All plants were placed in a growth chamber (Heraeus, Vötsch, Hanau, Germany) maintained at 25 °C in the daytime (16 h) and at 19 °C during the night (8 h). The humidity was maintained at 60%. 

After four weeks, the fresh clover was collected. The assessment of the effect of PAH and bacterial inoculation on the clover was based on the measurement of two parameters:

(1) The number of root nodules

(2) The dry weight of plants

The number of nodules was averaged over three plants in each combination (*n* = 3).

For the determination of the dry weight of the plants, collected clovers were dried at 55 °C for 24 h and then weighed (overground and underground parts of plants). The dry matter was measured with an accuracy of 0.0001 g and then averaged over each combination (*n* = 3).

### 2.5. Statistical Analysis 

Significant differences between *Rhizobium* sp. strains were calculated according to Fisher’s least significant difference (LSD) post-hoc test with significance level *P* ≤ 0.05 using Statistica.PL (version 10.0, StatSoft. Inc., Tulsa, OK, USA). 

To compare the relationship between the parameters obtained in the combination of plants inoculated with only *R. leguminosarum* strains and those co-inoculated with *A. brasilense*, the first step was to determine whether the distribution of the obtained results was similar to the normal distribution (Shapiro–Wilk test). The differences between the results in particular research groups were then analyzed. For this purpose, depending on the results of the Shapiro–Wilk test, the Mann–Whitney *U* test, Fisher’s test (to determine the similarity of variance), Student’s *t*-test or Student’s *t*-test with Welch’s correction was used. Correlation analyses were carried out with Spearman’s or Pearson’s correlation tests, depending on the Shapiro–Wilk test results. 

Spearman’s rank correlation coefficients were calculated to show the relations between observed clovers properties and doses of using PAHs.

All statistical analysis were carried out with use of R software packages (Northern Ave, Boston, MA, USA) and GraphPad Prism 8 (San Diego, CA, USA).

### 2.6. Data Visualisation

Heatmaps were generated using Gen III™ Omnilog values (data after 168 h of incubation) with R software (version 3.5.1, Northern Ave, Boston, USA) and pheatmap package. Similarity trees were constructed using Bray–Curtis cluster analysis with the UPGMA method [45].

Figures with statistical analysis were carried out with use of GraphPad Prism 8 (San Diego, CA, USA).

## 3. Results

### 3.1. Bacterial Species

As a result of the sequencing, it was determined that all eight sequences of *Rhizobium* have been assigned to one species, *Rhizobium leguminosarum*, with a sequence identity of 98.29–100%. The sequences of three strains of *Azospirillum* have been classified as *Azospirillum brasilense*, with a sequence identity of 100% (NCBI GenBank, Table 2).

### 3.2. Metabolic Activity of Rhizobium Leguminosarum Bacteria

**Figure 3:**
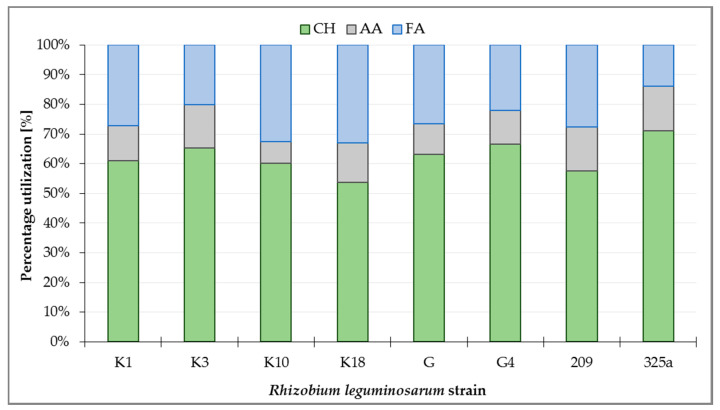
Percentage utilization of substrate groups by *Rhizobium leguminosarum* strains after 168 h incubation of GEN III™ plates: CH—carbohydrates; AA—amino acids; FA—fatty acids.

Metabolism analysis of all *R. leguminosarum* strains with the use of GEN III™ plates showed that carbohydrates (CH) were the most intensively utilized group of substrates, at a total level of about 63.3% (Figure 3). Analyzing the utilization of CH by *R. leguminosarum* strains, it can be observed that the 325a strain utilized compounds from this group the most intensively (71.05%), while the strain K18 was used the least (53.77%). Fatty acids (FA) were utilized at 13.85% (strain 325a) up to 33.01% (strain K18). The least utilized group of substrates by microorganisms were amino acids (AA), ranging from 7.28% (strain K10) to 15.11% (strain 325a, Figure 3).

**Figure 4:**
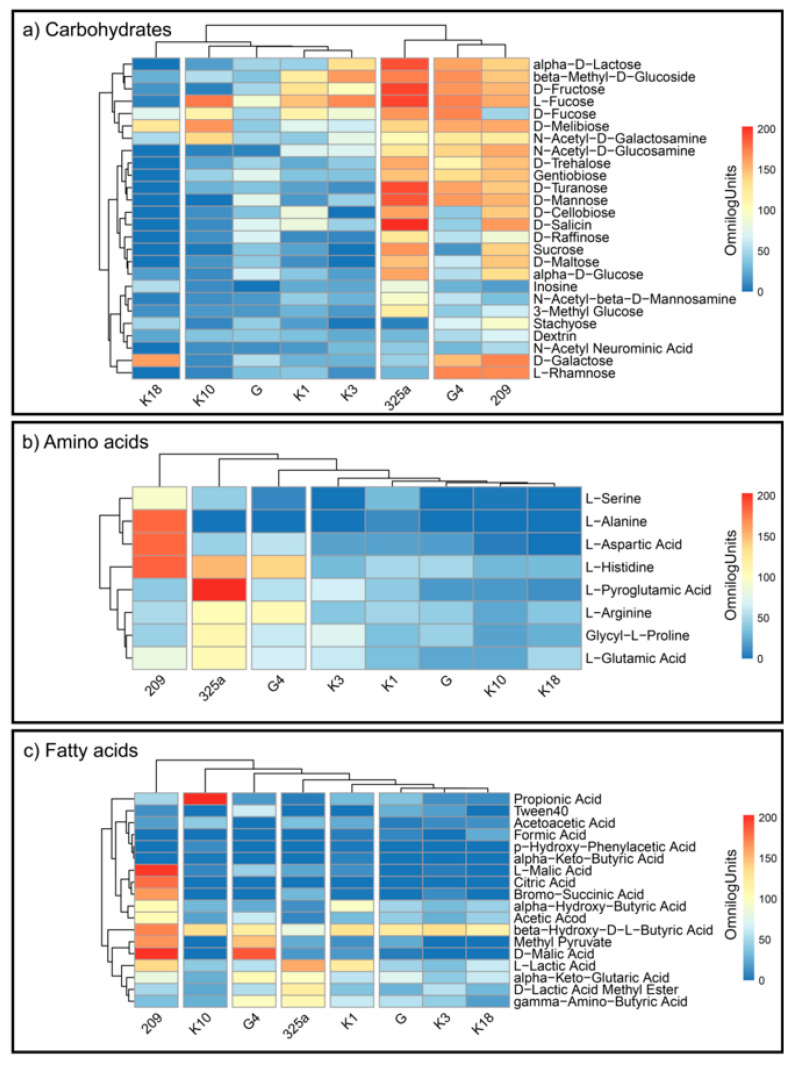
Heatmaps for the carbon utilization patterns of the substrates on GEN III™ grouped into three biochemical groups: (**a**) carbohydrates, (**b**) amino acids, and (**c**) fatty acids, by each strain of *R. leguminosarum*. Data are shown after 168 h of incubation. The gradient from light blue to red represents positive utilization.

*R. leguminosarum* strains decomposed the individual substrates at different levels (Figure 4a–c). This indicates a large metabolic diversity of the used strains. The analysis of CH utilization (Figure 4a) allowed us to divide *Rhizobium* strains into four groups: (1) K18; (2) K10, G, K1, K3; (3) 325a; and (4) G4 and 209 (Figure 4a). For the G4 and 209 strains, similar distribution of compounds from this group can be observed. The 325a strain has been intensely decomposed, among others, to alpha-D-Lactose, L-Fructose, and D-Fructose. An interesting observation is the low consumption of D-Fucose by strain 209 and intensive utilization of D-Galactose by strain K18, which generally showed low metabolic activity for compounds from the carbohydrates group.

By analyzing the differentiation of AA compounds degradation by *Rhizobium* strains, it was found that they were grouped into four main groups: (1) 209; (2) 325a; (3) G4; and (4) K3, K1, G, K10, K18 (Figure 4b). The most active growth (average) on media with AA was shown by strain 325a. At the same time, this strain slightly degraded L-alanine and L-aspartic acid, which in turn were intensively utilized by strain 209. L-Pyroglutamic Acid was preferred by strain 325a; interestingly, this compound was almost not decomposed by other strains. L-alanine was the least preferred substrate by all *R. leguminosarum* strains, except 209. Strain K10 showed the lowest average consumption of AA compared to other strains. 

Bacterial growth on media containing FA most varied the examined strains—six groups were obtained: (1) 209; (2) K10; (3) G4; (4) 325a; (5) K1; and (6) G, K3, K18 (Figure 4c). The strain with the most intensive FA metabolism was 209. K3 showed the lowest consumption of FA. The least degradable substrate overall was citric acid; however, strain 209 intensively metabolized this compound. An interesting observation is the distribution of propionic acid by the K10 strain. Other strains showed low or no decomposition of this compound, while K10 grew very intensively on medium with this substrate.

### 3.3. Biotest with Clover

The growth of clover on a medium contaminated with PAHs was analyzed by measuring the dry weight of plants and the number of root nodules after four weeks of plant growth on contaminated media.

#### 3.3.1. The Dry Weight of Clover

**Figure 5:**
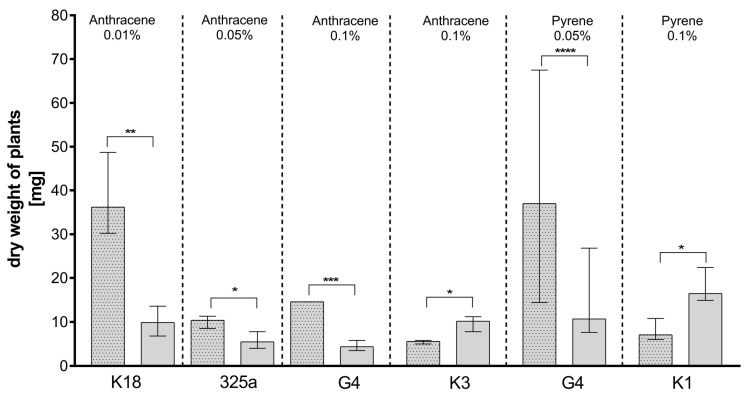
Comparison of the effect of inoculation of clover with only *R. leguminosarum* strains and with co-inoculation with *A. brasilense* on dry weight of clover (mg). The dotted posts indicate the co-inoculation with *A. brasilense* variant. The results are presented as a boxplot with median and 2.5–97.5 percentile. The symbols indicate significant results at: * *p* ≤ 0.05; ** *p* ≤ 0.01; *** *p* ≤ 0.001; **** *p* ≤ 0.0001; α = 0.05. Figure presents only those strains for which statistically significant differences were noted.

The dry weight of clover inoculated with *R. leguminosarum* strains only and co-inoculated with a mixture of *R. leguminosarum* strains and *A. brasilense* was calculated (Table 3 and Figure 5). 

Comparing the effect of individual strains on the dry weight of clover, it can be observed that in both combinations (single and mixed inoculation), different statistically significant results were obtained (Table 3). In the control, the highest dry weight was recorded for clover inoculated with single K10 and K18 strains, and K3 and 209 co-inoculated *Azospirillum* sp. (Table 3). 

For the plants growing in the presence of anthracene, the addition of the K10 and K3 strains allowed us to obtain a higher plant weight at a single *Rhizobium* inoculation (Table 3a). Interestingly, in case of *Azospirillum* co-inoculation and the K18 strain, a significant weight gain at 0.01% anthracene concentration was recorded.

Clover necrosis in 17 pots was recorded in case of phenanthrene contamination at 0.05 and 0.1% concentration (Table 3b). The highest number of plants died in the variant of co-inoculated *Azospirillum* in a medium contaminated with 0.1% phenanthrene. Comparing the effect of individual *Rhizobium* strains on the dry weight of clover at contamination with 0.01% phenanthrene, it can be seen that in single inoculation, significantly higher values were obtained for the K18 strain, compared to other strains. Single inoculation with the K10 strain of clover growing in the presence of pyrene allowed us to obtain statistically higher dry weight in the variant with 0.01% and 0.1% concentration and the G strain in the presence of 0.01% pyrene (Table 3c). In the variant of co-inoculation with *Azospirillum* sp., significantly higher dry weight values were noted for the G (0.01%) and G4 (0.05%) strains.

The presence of phenanthrene in the medium caused a very low increase in clover dry weight compared to the control plant (Table 3b). However, in the case of clover inoculated with strain K18, a higher dry weight of plants was observed in the presence of phenanthrene (0.01% dose) than in the control sample. The lowest values of dry plant weight were recorded at a dose of 0.1% phenanthrene. Co-inoculation with *A. brasilense* had a positive effect on plant dry matter at the lowest dose of phenanthrene (0.01%) in the case of strains 325a, 209, K1, and K3 of *R. leguminosarum*. At a dose of 0.05%, co-inoculation with both bacteria resulted in higher dry weights of clover in the presence of strains 209, G4, K10, and K18. In the case of the K18 strain, the difference in the obtained dry weight of clover was more than 90%. 

In the presence of pyrene, clover inoculated with strain K18 in the presence of *A. brasilense* (Table 3c) had a higher dry weight than the control sample. At a dose of 0.01% pyrene, co-inoculation with *A. brasilense* had a positive effect in the presence of strains K18, K1, G4, G, and 209. For strain 209, the increase in the dry weight of clover in the presence of *A. brasilense* was more than fourfold. At a pyrene dose of 0.05% and co-inoculated with two bacteria, clover had a higher dry weight using strains 209, 325a, G4, K1, and K18. At a pyrene dose of 0.1%, an increase in dry weight was only apparent in the presence of strains 325a and K3.

Analyzing the values of clover dry weight, it was observed that the values obtained between plants inoculated with strains of *R. leguminosarum* only and those co-inoculated with *A. brasilense* were significant statistically differentiated only in six variants (Figure 5). 

Analyzing the effect of co-inoculation on dry clover weight in the presence of anthracene contamination (Table 3a), it could be observed that at a dose of 0.01%, the dry weight of plants increased in seven out of eight cases. A slight decrease in dry weight after additional inoculation was observed with strain K3 only. At a dose of 0.05%, an increase in dry weight was also observed in seven cases after *A. brasilense* co-inoculation. A decrease was observed in the presence of the K10 strain. Plants inoculated with strains K10, K3, and 209 and co-inoculated with *A. brasilense* also showed lower dry weights on medium contaminated with 0.1% anthracene. Importantly, statistically significant differences were obtained only for four strains: K18, 325a, G4, and K3 (Figure 5). In the case of the K18 strain, in the presence of anthracene in doses of 0.01%, the addition of *A. brasilense* caused a statistical increase in the dry weight of plants. A statistical increase in the dry clover weight was also observed in combination of *A. brasilense* with the G4 and 325a strains in the presence of 0.05% and 0.1% of anthracene, respectively. Unexpectedly, with a dose of 0.1% of anthracene, the addition of *A. brasilense* to the K3 strain caused a statistically significant decrease in dry weight of clover.

In the presence of phenanthrene in the medium, the lowest values of dry plant weight were recorded at a dose of 0.1%. Co-inoculation with *A. brasilense* had a positive effect on plant dry matter at the lowest dose of phenanthrene (0.01%) in the case of strains 325a, 209, K1, and K3 of *R. leguminosarum*. At a dose of 0.05%, co-inoculation with both bacteria resulted in higher dry weights of clover in the presence of strains 209, G4, K10, and K18. In the case of the K18 strain, the difference in the obtained dry weight of clover was more than 90%. Unfortunately, the differences obtained are not statistically significant. The plant death in 17 variants makes a thorough comparative analysis difficult.

In the presence of pyrene, clover inoculated with strain K18 in the presence of *A. brasilense* (Table 3c) had a higher dry weight than the control sample. At a dose of 0.01% pyrene, co-inoculation with *A. brasilense* had a positive effect in the presence of strains K18, K1, G4, G, and 209. For strain 209, the increase in the dry weight of clover in the presence of *A. brasilense* was more than fourfold. At a pyrene dose of 0.05% and co-inoculated with two bacteria, clover had a higher dry weight using strains 209, 325a, G4, K1, and K18. At a pyrene dose of 0.1%, an increase in dry weight was only apparent in the presence of strains 325a and K3. However, statistically significant differences concerned only two variants. A statistical increase in the dry clover weight was also observed in combination of *A. brasilense* with the G4 strain in the presence of 0.05% of pyrene. The addition of *A. brasilense* to the K1 strain in the presence of pyrene at a dose of 0.1% was associated with a decrease in plant dry weight.

**Figure 6:**
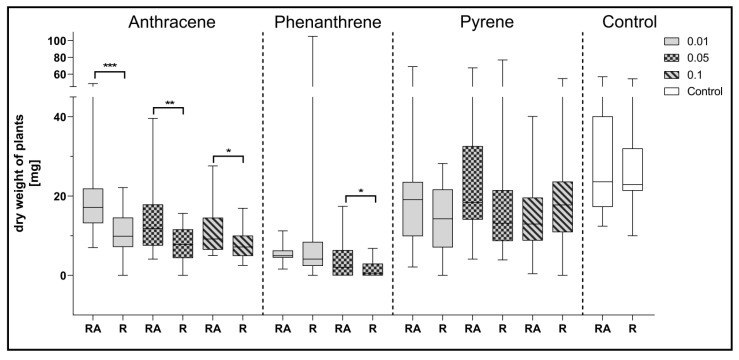
Comparison of the effect of inoculation of clover with only *R. leguminosarum* strains (R) and with co-inoculation with *A. brasilense* (RA) on average dry weight values of clover (mg). Control samples were grown without PAH. The results are presented as a boxplot with median and 2.5–97.5 percentile. The symbols indicate significant results at: * *p* ≤ 0.05; ** *p* ≤ 0.01; *** *p* ≤ 0.001; α = 0.05 (*n* = 24).

Comparing the effect of mixed inoculation with *A. brasilense* and *R. leguminosarum* with the inoculation with *R. leguminosarum* alone on the dry weight of clover, it can be noted that statistically significant differences were obtained for plants growing on a medium contaminated with all doses of anthracene and with 0.05% of phenanthrene concentration (Figure 6). 

#### 3.3.2. The Number of Root Nodules

The number of root nodules that grew on clover inoculated with *R. leguminosarum* strains alone and those co-inoculated with a mixture of *R. leguminosarum* strains and *A. brasilense* was counted (Table 4). The number of root nodules on clover differed depending on the strain of *R. leguminosarum* used and the dose and type of PAH. In the presence of phenanthrene, no nodules were obtained from these plants, or the mean of three plants was 0.0, despite clover growth (Table 3). Root nodules were only found in plants grown with a phenanthrene dose of 0.01% and co-inoculated with *A. brasilense* and the *R. leguminosarum* strains 325a and K3 (1.3 for 325a and 0.7 for strain K3, data not shown).

**Figure 7:**
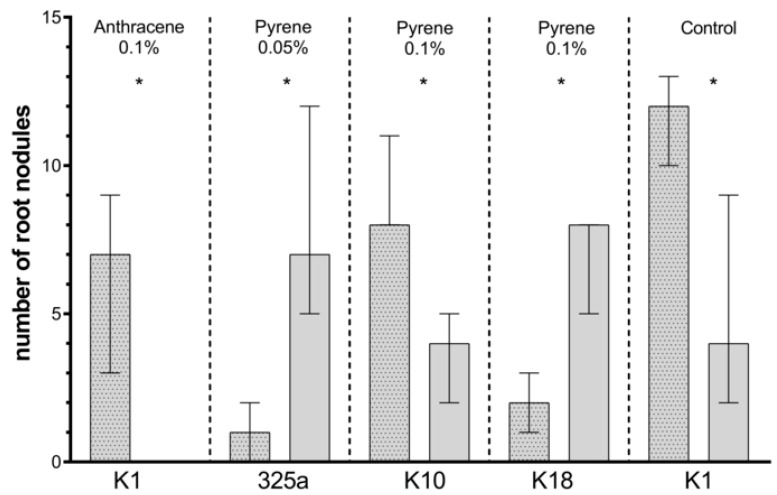
Comparison of the effect of inoculation of clover with only *R. leguminosarum* strains and with co-inoculation with *A. brasilense* on number of root nodules. The dotted posts indicate the co-inoculation with the *A. brasilense* variant. The results are presented as a boxplot with median and 2.5–97.5 percentile. The symbols indicate significant results at: * *p* ≤ 0.05; α = 0.05. Figure presents only those strains for which statistically significant differences were noted.

Comparing the number of root nodules obtained on clover in the presence of individual *Rhizobium* strains, it can be observed that the greatest variation was seen in combination with *Azospirillum*. In the presence of 0.05% anthracene, a statistically significantly higher number of root nodules was obtained with inoculation of a single K3 strain. In the variant with *Azospirillum*, the number of nodules was significantly higher in the presence of K3 (0.01%), K1 (0.05%), and 325a (0.1%) strains (Table 4a). Inoculation with 209, G, K1, and K18 strains in co-inoculation with *A. brasilense* had a positive effect on the number of root nodules in control samples (Table 4). However, only in the case of the K1 strain in the control, the addition of *A. brasilense* caused a statistical increase in the number of root nodules on clover (Figure 7).

On clover roots growing on a medium contaminated with anthracene, a higher number of nodules was observed in the presence of single *R. leguminosarum* inoculation with strain 209 at a dose of 0.01% and strain K3 at a dose of 0.05% (Table 4a). In other cases, the number of root nodules was higher with the use of co-inoculation with *A. brasilense*. At a dose of 0.1% anthracene, in the presence of six strains of *R. leguminosarum* (325a, G, G4, K1, K10, and K18), additional inoculation with *A. brasilense* increased the number of root nodules. Statistically significant differences concerned only the variant with K1 strain (Figure 7).

At a dose of 0.01% pyrene contamination, the positive effect of co-inoculation with *A. brasilense* on clover root nodules was observed in five cases: 209, G, G4, K3, and K10 (Table 4b). At a dose of 0.05% and 0.1%, the number of root nodules was higher in co-inoculated variants in four (209, K3, K10, and K18) and three (209, K3, and K10) strains of *R. leguminosarum*, respectively. A statistical increase in the number of nodules was observed in combination of *A. brasilense* with the K10 strain in the presence of pyrene at a dose of 0.1%. Unexpectedly, with a dose of 0.05% and 0.1% of pyrene, the addition of *A. brasilense* to the 325a and K18 strains caused a statistically significant decrease in the number of nodules (Figure 7).

**Figure 8:**
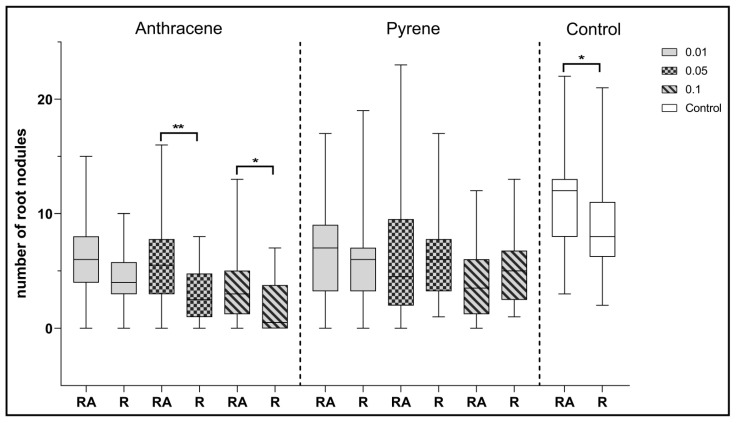
Comparison of the effect of inoculation of clover with only *R. leguminosarum* strains (*R)* and with co-inoculation with *A. brasilense* (RA) on the average number of root nodules. Control samples were grown without PAH. The results are presented as a boxplot with median and 2.5–97.5 percentile. The symbols indicate significant results at: * *p* ≤0.05; ** *p* ≤ 0.01; α = 0.05 (*n* = 24). No nodules were obtained from clover in combination with phenanthrene contamination.

Analyzing the effect of co-inoculation with *Azospirillum* and *Rhizobium* strains, it can be observed that statistically significant differences in the number of root nodules were observed in the control plants compared to clover inoculated with a single strain of *R. leguminosarum* (Figure 8). Statistically significant differences were also observed between the two inoculation combinations for clover growing on anthracene contaminated sand at 0.05 and 0.1%. In each of these examples, *Azospirillum* spp. co-inoculation had a positive effect on clover.

In both clover inoculation with single *R. leguminosarum* strains and with co-inoculation with *A. brasilense*, a statistically significant negative correlation was obtained between the dose of anthracene and the average dry weight values of plants, as well as the average number of root nodules (Table 5). The dose of phenanthrene correlated statistically significantly and strongly negatively (*P* ≤ 0.0001) with the average dry weight values of clover. Due to the lack of nodules on clover in this variant of contamination, no statistical analysis was performed for this parameter. Analyzing the correlation between the dose of pyrene and the parameters determined, a statistically significant negative correlation was observed with the average number of root nodules in the clover co-inoculated with *Azospirillum* sp.

## 4. Discussion

The *Pseudomonas* strain was isolated from *Trifolium pratense*, which degrades about 90% of phenanthrene and 7% of pyrene in the medium within seven days [10]. Furthermore, white clover (*T. repens*) produced root exudates, that promotes the dioxygenase gene copy number of *Mycobacterium*, which depredated PAHs [9]. Nevertheless, it has been reported that PAHs can be genotoxic for plants. For example, Aina et al. [4] showed that benzo[a]pyrene and naphthalene were genotoxic for white clover (*T. repens*) and induced changes in root and shoot DNA sequences. A negative effect of phenanthrene on the mycorrhization of red clover was also demonstrated [46]. The literature data indicate that white clover (*T. repens*) is highly sensitive to contamination with organic and inorganic compounds, which may also suggest the sensitivity of other plants of this type to PAHs such as the red clover (*T. pratense*) used in the presented experiment [4]. In the research presented here, a reduced dry weight of red clover in the presence of PAHs was recorded in comparison to the control (without contamination). At the same time, some studies indicate that PAH has no effect on the biomass of plants, including white and red clover [46]. Such different results are influenced by the type of substrate used (soil, sand) and the type and dose of PAH.

The increase in the number of clover root nodules observed in this research after the application of dual-culture inoculant (statistically significant only in three variants) is in accordance with the literature. In research conducted by Plazinski and Rolfe (1985), co-inoculation of white clover with isolates of *Rhizobium* and *Azospirillum* stimulated the formation of nodules by up to 25–100% [47]. The researchers have shown that the number of nodules depended on the type of *Azospirillum* strain used, not on *Rhizobium* [47]. This may indicate a positive symbiotic effect of these bacteria on nodulation. Our research indicates that individual *Rhizobium leguminosarum* strains have different effects on clover nodulation, both in single inoculation and co-inoculation with *Azospirillum brasilense*. Co-inoculation of guar *Azospirillum* and *Rhizobium* species resulted in an increase in the number of root nodules and dry weight of plants compared to inoculation with *Rhizobium* or *Azospirillum* alone [48]. Similar results were obtained for other plants, including pea, groundnut, and asparagus beans [49,50]. Co-inoculation of white clover with mixed inoculants of *A. lipoferum* and *R. leguminosarum* bv. *trifolii* inducted a threefold increase in nodulation compared with inoculation of plants with only *Rhizobium* spp. [51].

The results described in this paper have shown a large diversity between selected strains of *R. leguminosarum*, both in the metabolic aspect (consumption of substrate groups on Gen III Biolog) and clover dry weight obtained (regardless of the dose and type of PAH). Significant differences in the number of nodules formed on clover were found in the presence of anthracene and pyrene at a dose of 0.1%. After clover inoculation with *R. leguminosarum* strain K18, the highest values of plant dry weight were obtained, regardless of the type and dose of PAH. This may indicate that this strain promotes plant growth, e.g., by producing auxin (indole−3−acetic acid, IAA) and gibberellin GA_7_ [52]. At the same time, in the presence of this strain, the lowest number of root nodules was recorded in all variants of the experiment. Analyzing the metabolic activity (GEN III™ Biolog) of the examined *Rhizobium* strains, three strains can be distinguished: 325a, G4, and 209, which showed faster growth on GEN III™ plates, and three strains with slower growth: K18, K10, and K3. Furthermore, the K18 strain utilized carbohydrates media the least, while 325a and G4 very intensively decomposed compounds from this group. It could be assumed that active strains would have a more positive effect on plants. However, when analyzing the dry weight of clover, it can be observed that it was statistically higher than 325a, G4, and 209 when using the lower active K10 and K18 strains. On the other hand, in the case of the number of root nodules, the differences are not so visible. Will fast-growing strains intensively decomposing carbohydrates colonize the plants less readily while slower-growing strains establish the symbiosis that they need to replicate and grow? It is difficult to interpret such results, as there are no reports of the analysis of different strains of the same bacteria in the literature.

It has been demonstrated that the use of inoculants composed of *Azospirillum* and *Pseudomonas* strains have a positive effect on the root and shoot biomass of meadow fescue in the presence of PAH [53]. Research indicates that the rates of crude oil degradation and microbiological activity increase when the fungus *Scedsporium boydii* are added to the indigenous bacterial consortium [54]. Similar results were obtained for soils contaminated with 2,4-dinitrotoluene (DNT). A consortium of seven species of bacteria significantly increased the root length of *Arabidopsis*, despite the stress of 2.4-DNT [55]. For salt stress, inoculation with the *Rhizobium* consortium had a more positive effect on maize size, grain yield, protein content, and chlorophyll content compared to single strain inoculation [56]. All these data confirm that the synergic effects of microbial consortia have a greater effect on plant growth, plant quality, and soil micro- and/or phytoremediation from different contaminants. In our experiment, the contamination of the medium with phenanthrene caused a decrease in the number of root nodules in the case of plants only inoculated with *Rhizobium* spp. In the presented research, the effect of *Azospirillum* spp. co-inoculation of clover was analyzed, and statistically significant higher values of plant dry weight than after the application of *Rhizobium* spp. alone in four variants were observed. There was also an increase in the number of root nodules in three experimental variants. After the addition of *Azospirillum* spp., the appearance of root nodules was observed in the presence of the 325a and K3 strains. This may be a sign of the positive influence of these strains with *Azospirillum* spp. in phenanthrene presence. An increase in dry weight of clover was observed after application of the 325a, G4, and K18 strains in a mixture with *A. brasilense* strains in the presence of anthracene. The results also showed a negative effect of higher doses of PAH on average dry weight and root nodules values of clover inoculated only with *R. leguminosarum*.

The positive effect of co-inoculation of clover and lucerne with *Rhizobium* and *Azospirillum* strains on the process of free nitrogen fixation, as well as on plant yield, has been demonstrated by other researchers [57]. The stimulating effect of co-inoculation of *A. brasilense* in combination with *Rhizobium* sp. on yield, nodulation, and diazotrophs were also observed in the case of soya and bean seed [58] and maize [51]. It has been found that the presence of *Rhizobium* bacteria stimulates the colonization of maize roots by *Azospirillum*. At the same time, it has been shown that in the presence of *Azospirillum* and as a result of the phytohormones they produce, plants are more susceptible to infections with nodulation bacteria, including *Rhizobium* [59]. However, the analysis of individual variants does not allow such optimistic conclusions. *Azospirillum* co-inoculation had a statistically significant positive effect on the dry weight of clover only in four variants of the experiment and on nodulation in three cases. Furthermore, the K18 and 325a strains, in combination with *Azospirillum*, allowed us to obtain a statistically higher dry weight of clover, but at the same time, a statistically lower number of root nodules was observed than in single inoculation. Literature data showed positive correlations between nodule formation and biomass production [51,60]. However, Burton [61] found that the nodule number is not a good index of the benefit that the plant is receiving on clovers. Thus, a higher plant weight does not have to correlate positively with the number of nodules and vice versa, which corresponds to the data obtained in this experiment. This is an important aspect for further analysis. Those strains of *Rhizobium* (K1, K10, K18, G4, and 325a) which showed some positive effect in combination with *Azospirillum* can be further investigated in detail. The next step of the experiment will be the characterization of individual *R. leguminosarum* strains and the search for the most effective one. It would appear that the use of a dual-culture inoculant may have a positive impact on the nodulation of plants in the presence of contamination in the environment.

## 5. Conclusions

1) The dose of anthracene significantly negatively correlated with average dry weight values of clover and the average number of root nodules in both cases of inoculation.
2) Inoculation with strain K18 caused the highest values of the dry weight of clover and the number of root nodules compared to other strains, with low metabolic activity determined with GEN III™ plates.
3) The addition of phenanthrene to the medium had a negative effect on the formation of root nodules in clover inoculated only with *R. leguminosarum*.
4) Co-inoculation with *Azospirillum* had a statistically significant positive effect on the dry weight of clover in the medium contaminated with all doses of anthracene and on the number of root nodules at 0.05% and 0.1% anthracene.
5) The applied contamination with phenanthrene proved to be a factor limiting the formation of root nodules on clover.
6) Future research may include an analysis of *Rhizobium* spp. strains for bioremediation and an analysis of PAH distribution in the medium in the applied experimental scheme.

## Figures and Tables

**Table 1:** Polycyclic aromatic hydrocarbons used to contaminate sand in the biotest.

Characteristics	Anthracene	Phenanthrene	Pyrene
Chemical formula	C_14_H_10_	C_14_H_10_	C_16_H_10_
Number of aromatic rings	3	3	4
Molar mass (g mol^−1^)	178.23	178.23	202.25
Boiling point (°C)	339.9	332.0	404.0
Solubility in water (µg L^−1^ at 25 °C)	45	1300	132
TEF	0.01	0.001	0.001
Log *P*_oct/wat_	4.54	4.57	5.18

TEF: toxic equivalency factor, based on the toxicity of benzo[a]pyrene; Log *P*_oct/wat_: partition-coefficient octanol/water [42,43].

**Table 2:** Data on the identification of bacteria strains used in this experiment.

Abbreviation	GenBank Accession Number	Closest Species	Identity (%)	Coverage (%)
*Rhizobium* sp.				
209	MT605962	*Rhizobium leguminosarum*	100	100
325a	MT605963	*Rhizobium leguminosarum*	98.67	100
G	MT605964	*Rhizobium leguminosarum*	100	100
G4	MT605965	*Rhizobium leguminosarum*	100	100
K1	MT605966	*Rhizobium leguminosarum*	98.29	100
K3	MT605967	*Rhizobium leguminosarum*	100	100
K10	MT605968	*Rhizobium leguminosarum*	100	100
K18	MT605969	*Rhizobium leguminosarum*	99.91	100
*Azospirillum* sp.				
Azo1	MT814302	*Azospirillum brasilense*	100	100
Azo2	MT814301	*Azospirillum brasilense*	100	99.85
Azo3	MT814300	*Azospirillum brasilense*	100	100

All sequences are available at the NCBI database under accession number: SUB7603645.

**Table 3:** The dry weight of clover (mg) depending on the dose of contamination with each PAH: (**a**) anthracene; (**b**) phenanthrene; (**c**) pyrene. The inoculated combination is given by *R* (inoculated with *Rhizobium leguminosarum* only) or *RA* (co-inoculated with *Rhizobium leguminosarum* and *Azospirillum brasilense* strains). Control samples were grown without PAH. The different letters (a–d) in the columns indicate significant results (Fisher’s LSD test, *P* ≤ 0.05, *n* = 3). * indicates that in this combination, the average dry weight of the plants (*n* = 3) was 0.0.

(a)		Anthracene							
Inoculated Combination		*R*	*RA*	*R*	*RA*	*R*	*RA*	*R*	*RA*
PAH Doses		**0.01%**		**0.05%**		**0.1%**		**Control**	
Strain	**209**	7.83 ^b^	10.10 ^c^	4.27 ^b^	8.30 ^b^	9.40 ^ab^	8.77 ^ab^	25.17 ^ab^	37.80 ^a^
	**325a**	6.73 ^b^	16.33 ^cb^	5.77 ^b^	10.07 ^b^	6.00 ^ab^	11.34 ^ab^	24.93 ^ab^	19.87 ^ab^
	**G**	13.04 ^ab^	19.00 ^cb^	7.67 ^ab^	21.70 ^ab^	5.80 ^b^	15.13 ^a^	13.70 ^b^	33.97 ^ab^
	**G4**	6.70 ^b^	14.60 ^cb^	5.80 ^b^	10.33 ^b^	4.57 ^b^	14.60 ^ab^	33.63 ^a^	25.57 ^ab^
	**K1**	10.80 ^ab^	15.67 ^cb^	8.90 ^ab^	14.60 ^ab^	6.03 ^ab^	10.50 ^ab^	23.57 ^ab^	14.60 ^b^
	**K3**	16.90 ^a^	16.77 ^cb^	13.33 ^a^	25.17 ^a^	9.73 ^ab^	5.47 ^b^	22.90 ^ab^	40.83 ^a^
	**K10**	17.03 ^a^	21.50 ^b^	8.57 ^ab^	7.87 ^b^	11.70 ^a^	11.63 ^ab^	39.70 ^a^	23.43 ^ab^
	**K18**	10.10 ^ab^	38.37 ^a^	10.20 ^ab^	15.93 ^ab^	7.97 ^ab^	8.77 ^ab^	32.27 ^a^	30.13 ^ab^
**(b)**		**Phenanthrene**							
Inoculated Combination		***R***	***RA***	***R***	***RA***	***R***	***RA***	***R***	***RA***
PAH Doses		**0.01%**		**0.05%**		**0.1%**		**Control**	
Strain	**209**	2.20 ^b^	4.93 ^b^	3.33 ^ab^	6.30 ^bc^	0.00 * ^a^	0.00 * ^b^	25.17 ^ab^	37.80 ^a^
	**325a**	3.50 ^ab^	7.87 ^a^	0.00 * ^c^	0.00 * ^d^	0.00 * ^a^	3.20 ^a^	24.93 ^ab^	19.87 ^ab^
	**G**	6.17 ^ab^	2.20 ^c^	4.57 ^a^	0.00 * ^d^	0.00 * ^a^	0.00 * ^b^	13.70 ^b^	33.97 ^ab^
	**G4**	12.63 ^ab^	4.80 ^b^	2.57 ^abc^	8.40 ^ab^	2.27 ^a^	0.00 * ^b^	33.63 ^a^	25.57 ^ab^
	**K1**	4.40 ^ab^	8.20 ^a^	2.73 ^ab^	2.00 ^d^	0.00 * ^a^	0.00 * ^b^	23.57 ^ab^	14.60 ^b^
	**K3**	4.97 ^ab^	5.17 ^b^	0.00 * ^c^	0.00 * ^d^	1.07 ^a^	0.00 * ^b^	22.90 ^ab^	40.83 ^a^
	**K10**	4.20 ^ab^	4.13 ^bc^	0.00 * ^c^	2.70 ^cd^	1.07 ^a^	0.00 * ^b^	39.70 ^a^	23.43 ^ab^
	**K18**	38.83 ^a^	6.00 ^ab^	0.83 ^bc^	11.10 ^a^	0.93 ^a^	0.00 * ^b^	32.27 ^a^	30.13 ^ab^
**(c)**		**Pyrene**							
Inoculated Combination		***R***	***RA***	***R***	***RA***	***R***	***RA***	***R***	***RA***
PAH Doses		**0.01%**		**0.05%**		**0.1%**		**Control**	
Strain	**209**	4.93 ^b^	20.43 ^ab^	10.23 ^a^	24.73 ^ab^	22.83 ^abc^	11.80 ^a^	25.17 ^ab^	37.80 ^a^
	**325a**	15.00 ^ab^	11.53 ^ab^	12.87 ^a^	16.10 ^ab^	8.73 ^c^	10.40 ^a^	24.93 ^ab^	19.87 ^ab^
	**G**	22.23 ^a^	36.43 ^a^	18.40 ^a^	15.57 ^b^	16.27 ^abc^	15.40 ^a^	13.70 ^b^	33.97 ^ab^
	**G4**	6.13 ^b^	15.70 ^ab^	15.03 ^a^	39.63 ^a^	17.03 ^abc^	12.83 ^a^	33.63 ^a^	25.57 ^ab^
	**K1**	15.50 ^ab^	24.07 ^ab^	9.50 ^a^	15.13 ^b^	17.93 ^abc^	7.97 ^a^	23.57 ^ab^	14.60 ^b^
	**K3**	12.60 ^ab^	9.90 ^b^	33.90 ^a^	24.73 ^ab^	11.83 ^bc^	16.43 ^a^	22.90 ^ab^	40.83 ^a^
	**K10**	19.87 ^a^	15.50 ^ab^	24.03 ^a^	23.30 ^ab^	34.33 ^a^	22.43 ^a^	39.70 ^a^	23.43 ^ab^
	**K18**	14.93 ^ab^	35.93 ^ab^	21.27 ^a^	25.63 ^ab^	30.93 ^ab^	22.67 ^a^	32.27 ^a^	30.13 ^ab^

**Table 4:** Average (*n = 3*) number of root nodules on clover depending on the dose of contamination with each PAH: (**a**) anthracene and (**b**) pyrene. The inoculated combination is given by *R* (inoculated with *R. leguminosarum* only) or *RA* (co-inoculated with *R. leguminosarum* and *A. brasilense* strains). Control samples were grown without PAH. The different letters (a–c) in the columns indicate significant results (Fisher’s LSD test, *P* ≤ 0.05, *n* = 3). * means that in this combination the average number of root nodules on the plants (*n* = 3) was 0.0.

(a)		Anthracene							
Inoculated Combination		*R*	*RA*	*R*	*RA*	*R*	*RA*	*R*	*RA*
PAH Doses		**0.01%**		**0.05%**		**0.1%**		**Control**	
Strain	**209**	5.7 ^a^	3.0 ^b^	1.0 ^b^	4.7 ^ab^	4.3 ^a^	1.7 ^bc^	11.3 ^a^	12.3 ^b^
	**325a**	5.0 ^a^	8.0 ^ab^	1.7 ^ab^	5.3 ^ab^	4.3 ^a^	7.3 ^a^	11.3 ^a^	11.3 ^b^
	**G**	4.0 ^a^	6.7 ^ab^	1.7 ^ab^	8.0 ^ab^	1.0 ^ab^	2.7 ^abc^	11.7 ^a^	19.3 ^a^
	**G4**	4.3 ^a^	5.3 ^ab^	2.7 ^ab^	5.0 ^ab^	0.0 *^b^	5.0 ^ab^	10.7 ^a^	10.0 ^b^
	**K1**	2.0 ^a^	3.7 ^b^	4.3 ^ab^	11.0 ^a^	0.0*^b^	6.3 ^ab^	5.0 ^a^	11.7 ^b^
	**K3**	6.0 ^a^	10.3 ^a^	5.3 ^a^	3.0 ^b^	3.0 ^ab^	0.0 *^c^	9.3 ^a^	8.7 ^b^
	**K10**	3.3 ^a^	6.0 ^ab^	3.0 ^ab^	3.3 ^b^	1.0 ^ab^	6.0 ^ab^	10.0 ^a^	9.7 ^b^
	**K18**	4.0 ^a^	5.0 ^ab^	3.3 ^b^	6.3 ^ab^	0.7 ^b^	2.7 ^abc^	6.0 ^a^	10.0 ^b^
**(b)**		**Pyrene**							
Inoculated Combination		***R***	***RA***	***R***	***RA***	***R***	***RA***	***R***	***RA***
PAH Doses		**0.01%**		**0.05%**		**0.1%**		**Control**	
Strain	**209**	9.3 ^a^	10.3 ^a^	8.0 ^a^	12.7 ^a^	2.0 ^c^	4.0 ^b^	11.3 ^a^	12.3 ^b^
	**325a**	9.3 ^a^	7.0 ^abc^	8.0 ^a^	1.0 ^b^	2.3 ^c^	2.0 ^b^	11.3 ^a^	11.3 ^b^
	**G**	6.3 ^a^	8.7 ^abc^	4.7 ^a^	1.0 ^b^	4.0 ^bc^	2.0 ^b^	11.7 ^a^	19.3 ^a^
	**G4**	2.3 ^a^	2.7 ^c^	5.3 ^a^	5.3 ^ab^	7.3 ^ab^	2.7 ^b^	10.7 ^a^	10.0 ^b^
	**K1**	8.0 ^a^	7.7 ^abc^	4.7 ^a^	4.7 ^ab^	9.7 ^a^	5.3 ^ab^	5.0 ^a^	11.7 ^b^
	**K3**	4.0 ^a^	5.0 ^abc^	8.7 ^a^	10.3 ^a^	4.3 ^bc^	6.0 ^ab^	9.3 ^a^	8.7 ^b^
	**K10**	7.7 ^a^	9.7 ^ab^	6.3 ^a^	7.3 ^ab^	3.7 ^bc^	9.0 ^a^	10.0 ^a^	9.7 ^b^
	**K18**	4.0 ^a^	3.3 ^bc^	4.0 ^a^	6.0 ^ab^	7.0 ^bc^	2.0 ^b^	6.0 ^a^	10.0 ^b^

**Table 5:** Spearman’s correlation coefficients between observed parameters (average) of red clover and doses of PAHs: anthracene, phenanthrene, and pyrene for eight using strains of *Rhizobium*. Significant levels for the Spearman’s rank coefficients are indicated at the * *p* ≤0.05 and **** *p* ≤ 0.0001 levels.

		Anthracene Doses		Phenanthrene Doses		Pyrene Doses	
Parameter	Inoculation	*r*	*p*	*r*	*p*	*r*	*p*
Dry weight of clover	***R. leguminosarum***	−0.2902	0.0134 *	−0.6256	<0.0001 ****	0.1993	0.0932
	***R. leguminosarum + A. brasilense***	−0.4593	<0.0001 ****	−0.6852	<0.0001 ****	−0.1658	0.1641
Number of root nodules	***R. leguminosarum***	−0.4180	0.0003 *	−	−	−0.1305	0.2746
	***R. leguminosarum + A. brasilense***	−0.2635	0.0253 *	−	−	−0.2720	0.0208 *
